# Supplementary material for: Magnetization reversal driven by low dimensional chaos in a nanoscale ferromagnet
Source: Nat Commun. 2019 Feb 1;10:543. doi: 10.1038/s41467-019-08444-2 (PMC6358601; doi:10.1038/s41467-019-08444-2)
Supplement: Supplementary file 1 — Supplementary Information [file 41467_2019_8444_MOESM1_ESM.pdf]

Supplementary Information

Magnetization reversal driven by low dimensional chaos in a nanoscale ferromagnet

Montoya et al.

SUPPLEMENTARY NOTE 1: SPIN TORQUE FERROMAGNETIC RESONANCE

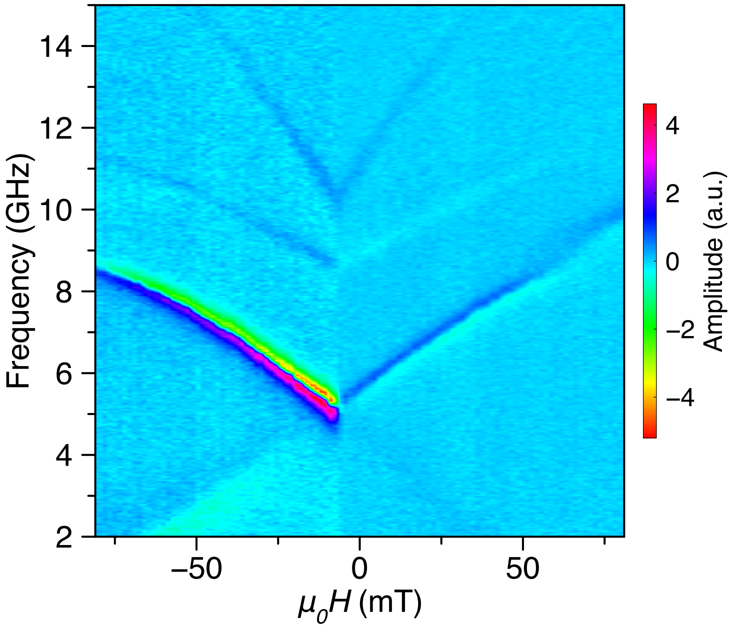

Supplementary Figure 1. **Spin torque ferromagnetic resonance measurement.**

We employ field-modulated spin torque ferromagnetic resonance (ST-FMR) technique to determine the ferromagnetic resonance frequency f_{FMR} in our samples[1,2]. In these measurements, microwave voltage is applied to the MTJ through the ac port of the bias tee, and rectified voltage generated by the MTJ at the frequency of magnetic field modulation is measured by a lock-in amplifier through the DC port of the bias tee, as schematically illustrated in Fig. 2a of the main text. The ST-FMR spectra measured as a function of in-plane magnetic field applied parallel to the free layer easy axis reveal that the lowest frequency mode of the free layer (the quasi-uniform FMR mode) in zero applied field is $f_{\mathrm{FMR}} = 5.1$ GHz, as shown in Supplementary Figure 1[1]. A higher order spin wave mode is observed at $f = 9$ GHz[1]. In the main text we are mainly concerned with the magnetization dynamics at driving frequencies below f_{FMR}.

SUPPLEMENTARY NOTE 2: STOCHASTIC RESONANCE

Stochastic resonance is a phenomenon observed in nonlinear systems, by which a low amplitude periodic input signal is amplified by noise. The stochastic resonance effect requires a given system to have three basic components[3]: (i) an energy barrier separating stable equilibria, (ii) a (weak) coherent input driving the system dynamics, (iii) a source of noise. AC driven nanomagnets at finite temperature, such as those studied in this article, have these components and have been shown to display stochastic resonance[4–6]. In the free layer of our system, the uniaxial magnetic anisotropy provides the energy barrier (i), while thermal fluctuations provide the source of noise (iii). The coherent drive (ii) is provided by the ac spin transfer torque. In this Supplementary Note, we report measurements of stochastic resonance in MTJ devices studied in this work in order to understand the relation of this effect to the chaos-induced switching data studied in this article.

We perform stochastic resonance measurements on an MTJ sample, which dimensions are nominally identical to that studied in the main text. We make measurements at the external field required to balance the dwell times in the P and AP states ($\tau = \tau_{\mathrm{P}} = \tau_{\mathrm{AP}}$) at room temperature ($T = 300\,\mathrm{K}$) and zero direct current, and find the switching rate to be $w = (1/\tau) = 767$ Hz. Literature on stochastic resonance in bistable systems[7–9] usually deals with the Kramers rate of particle switching from one state to the other and back $r_{\mathrm{K}} = 1/\left(\tau_{\mathrm{P}} + \tau_{\mathrm{AP}}\right) = 1/2\tau = \omega/2 = 383.5$ Hz.

We apply an ac current $I_{\mathrm{ac}}(t)$ to a circuit consisting of the MTJ in series with a 101.2 kΩ resistor and measure the real-time voltage $V(t)$ across the MTJ with a high performance DAQ as a function of ac drive frequency f as illustrated in Supplementary Figure 2 (top and middle panels). We then calculate the resistance of the MTJ as a

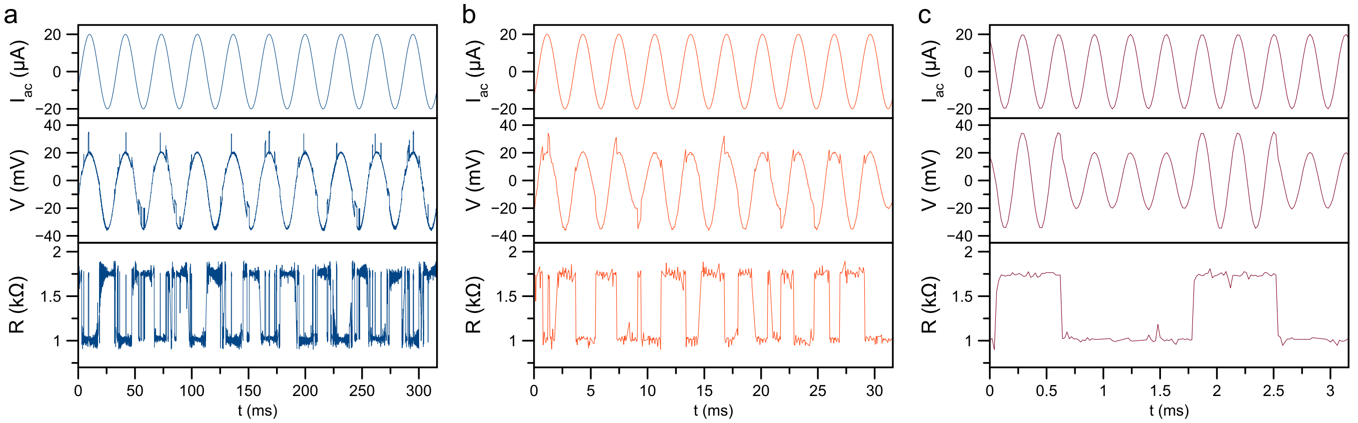

Supplementary Figure 2. **Room temperature real-time stochastic resonance measurements.** Stochastic resonance data measured for three values of the drive frequency f: **a** 31.6 Hz, **b** 316.2 Hz and **c** 3162.3 Hz. In **a-c**, the top panel shows the ac drive current, the middle panel is the measured voltage, and the bottom panel is resistance calculated by dividing the measured voltage by the current.

function of time $R(t) = V(t)/I_{\rm ac}(t)$ to directly monitor random telegraph noise of the MTJ free layer switching between the P and AP states as shown in the bottom panels of Supplementary Figure 2. Short time intervals where $|I_{\rm ac}| < 1\mu$A lead to low-bias division anomalies; therefore, in these regions, we employ linear interpolation of the $R(t)$ data between measured resistance values at the edges of these short time intervals. Supplementary Figure 2a shows example switching characteristics for ac drive frequency of $f = 31.6$ Hz. In this regime, the drive frequency is much below the Kramers rate, $f \ll r_{\rm K}$, and we observe a significant degree of synchronization of the system state given by $R(t)$ to the ac spin torque drive due to $I_{\rm ac}(t)$: when $I_{\rm ac} > 0$ the device has large probability to be in the P-state, while for $I_{\rm ac} < 0$ the device prefers the AP-state. However, a number of thermally activated brief excursions into the opposite state still occur during the half cycle of the ac drive. Supplementary Figure 2b shows behavior for drive frequency $f = 316.2$ Hz $\approx r_{\rm K}$. In this regime, there is a moderate degree of entrainment of the system state to the ac drive. Supplementary Figure 2c shows behavior for drive frequency $f = 3162.3 \gg r_{\rm K}$. In this regime, there is negligible entrainment. The observed dependence of the degree of entrainment on the ac drive frequency is a telltale signature of stochastic resonance (see, for example, Fig. 4b of the review article by Gammaitoni et al.[7]).

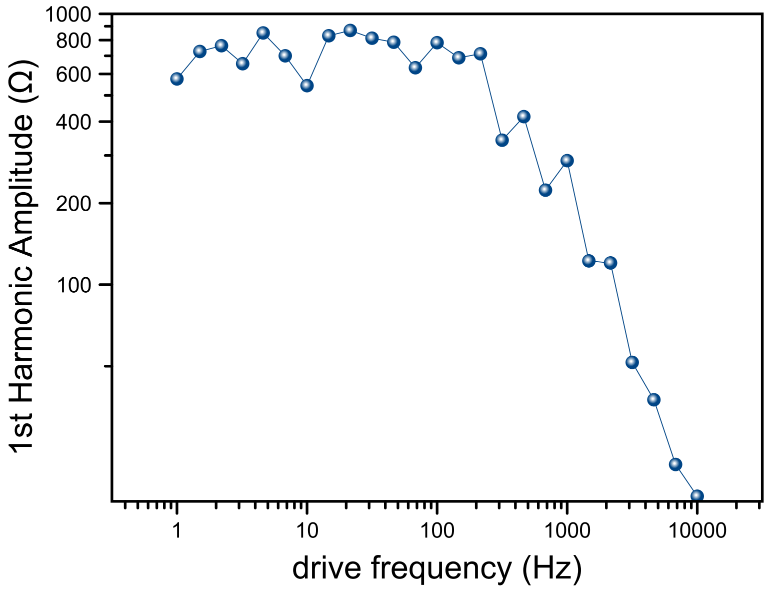

Supplementary Figure 3. **Frequency dependence.** The amplitude of the 1st harmonic FFT response as a function of ac drive frequency f.

The stochastic resonance behavior can be further confirmed via taking the fast Fourier transform (FFT) of $R(t)$ and plotting the first harmonic of the FFT response (the value of FFT at the frequency equal to the drive frequency) as a function of the drive frequency, as illustrated in Supplementary Figure 3. The first harmonic response gives a measure of the entrainment of the MTJ state to the ac drive. For $f < r_{\mathrm{K}}$ the amplitude of the first harmonic is relatively large and constant as the MTJ free layer has sufficient time to switch into the favorable state during half-period of the ac drive. When $f \approx r_{\mathrm{K}}$, the first harmonic amplitude begins to decay rapidly with increasing f. This reflects the fact the thermally activated switching rate r_{K} becomes too low for the free layer to follow the ac drive. The frequency dependence of the first harmonic amplitude shown in Supplementary Figure 3 is another salient feature of stochastic resonance (see, for example, Fig. 11 of Ref. [7]). We note that the free layer of the MTJ in the main text exhibits Kramers rate $r_{\mathrm{K}} = 36$ Hz. This means that the data presented in the main text is all in the regime where $f \gg r_{\mathrm{K}}$, and therefore stochastic resonance effect cannot explain the influence of ac drive on stochastic switching of the free layer magnetization described in the main text.

The chaotic process that leads to magnetization reversal in our system at frequencies much greater than r_{K} requires two of the components required for stochastic resonance: (i) energy barrier that leads to threshold behavior, (ii) coherent input provided by microwave spin transfer torque. The chaotic process is, however, distinctly different from stochastic resonance as it does not require (iii) a source of noise. In fact, it is remarkable that the observed chaotic dynamics are robust to thermal noise.

SUPPLEMENTARY NOTE 3: BASIN EROSION

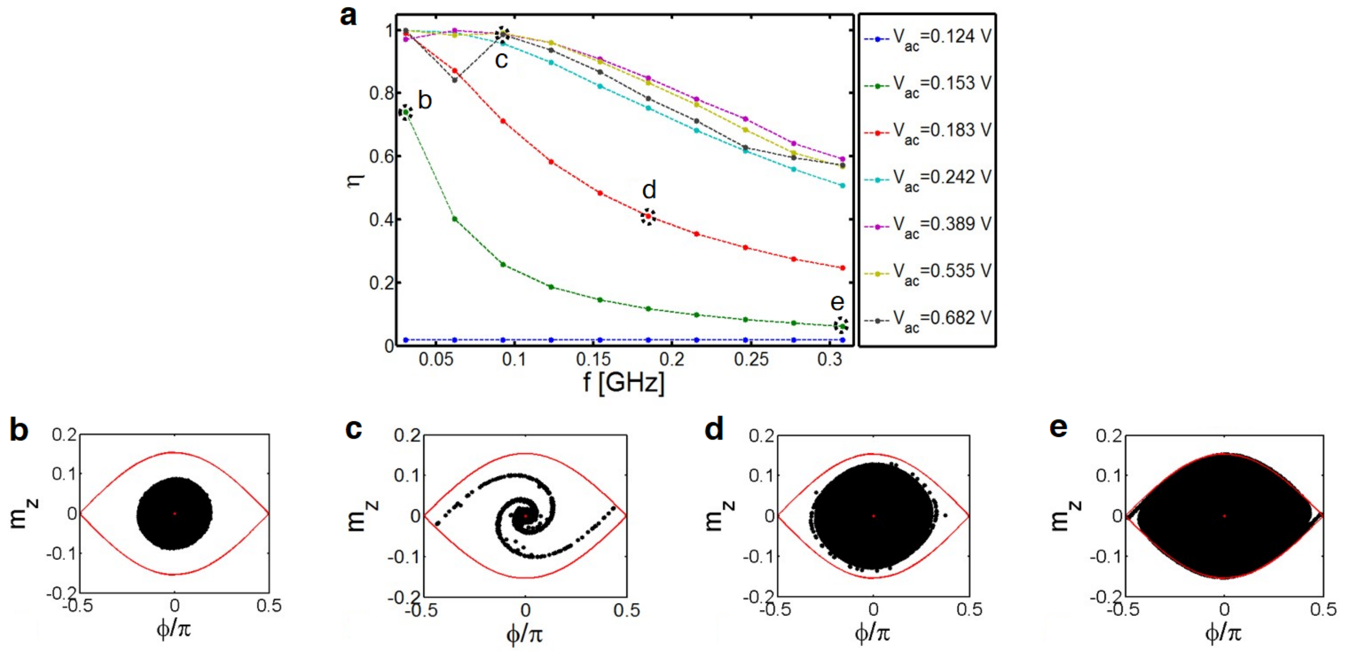

Supplementary Figure 4. **Quantitative analysis of basin erosion.** Top panel a: degree of erosion η as a function of ac drive frequency f and voltage V_{ac}. Lower panels b,c,d,e: numerically computed basin erosion produced in the (ϕ, m_z)-plane for $T = 0$ and ac spin-torque polarizer along the x direction. The low energy well is initially filled by $N = 10^5$ phase points. When the trajectory originating from a phase point escapes the well within $n = 5$ iterations of the map (main text equation (3)), it is considered 'unsafe' and disregarded. The remaining phase points correspond to 'safe' initial conditions. Panels b,c,d,e refer to different ac frequency/amplitude pairs (associated with markers b,c,d,e in top panel a). Values of parameters refer to a MTJ with $75 \times 50 \times 1.8$ nm^3 elliptical free layer for which $\mu_0 M_{\mathrm{s}} = 1.1T, D_x = 0.035, D_y = 0.056, D_z = 0.909, \alpha = 0.016$. Dimensionless angular frequency $\omega = 1$ corresponds to frequency $f = \gamma M_{\mathrm{s}}/(2\pi) = 30.789$ GHz, dimensionless ac spin-torque $\beta_{\mathrm{ac}} = 1$ corresponds to injected ac current with amplitude $I_{\mathrm{ac}} = J_{\mathrm{p}} S/(2\lambda) = 6.5$ mA (polarization factor $\lambda = 0.6$, cross-sectional MTJ area $S = 2.9452 \times 10^{-15}$ m^2, $J_{\mathrm{p}} = |e|\gamma M_{\mathrm{s}}^2 t_{\mathrm{FL}}/(g_{\mathrm{L}}\mu_{\mathrm{B}}) = 2.63 \times 10^{12}$ Am^{-2}, $t_{\mathrm{FL}} = 1.8$ nm). Conversion between current and voltage applied to the MTJ is performed as $V_{\mathrm{ac}} = RI_{\mathrm{ac}}$ where the resistance $R = (R_{\mathrm{P}} + R_{\mathrm{AP}})/2 = 2400\Omega$ is the average between the measured MTJ resistance values in the parallel and anti-parallel states (see section Methods); thus, a value of the dimensionless ac spin-torque $\beta_{\mathrm{ac}} = 1$ corresponds to an applied voltage $V_{\mathrm{ac}} = 2400\Omega \times 6.5\mathrm{mA} = 15.5$ V.

A direct consequence arising from the onset of chaotic magnetization dynamics is the phenomenon of erosion of the

basins of attraction of asymptotic regimes[3], i.e. regions of magnetization dynamics that stay within a given energy well for arbitrarily long times. In fact, the effect of the heteroclinic tangle (chaotic saddle) and the presence of lobes lead to the deterministic escape of magnetization trajectories within the potential well around one free energy minima to outside the well. The erosion has important practical consequences as it is similar to a reduction of the depth of the potential well. Thus, it reduces the 'safety region' around a stable equilibrium state. In addition, the boundary of the basin of attraction of asymptotic regimes inside the well acquires a fractal nature.

We stress that the basin erosion phenomenon is of purely deterministic origin and can be studied numerically[10,11] by iterating the stroboscopic map (main text equation (3)) associated with zero-temperature LL equation for an ensemble of a very large number N of initial conditions filling one energy well and progressively removing the states that escape the energy well. In this section, we will refer to the energy well around $\boldsymbol{m} = +\boldsymbol{x}$. Quantitative analysis of the basin erosion can be performed by defining the degree of erosion η as the fraction of particles in the ensemble which escape the well in a given number n of iterates of the stroboscopic map[11]. We have performed extensive computations of η as function of ac voltage and frequency. The results are reported in Supplementary Figure 4a. The zero temperature simulations are carried out for $N = 10^5$ ensemble particles and $n = 5$ iterates of the stroboscopic map, and with ac spin-torque polarizer directed along the easy anisotropy axis x. Under this ac drive configuration, the ferromagnetic resonance of the nanoparticle is inhibited and, consequently, the excitation of chaotic dynamics is not disturbed by the interplay with magnetic resonance phenomena. Moreover, We have checked that choosing $n > 5$ does not significantly affect the results, which can be ascribed to the aforementioned fractal nature of the lobe dynamics[12].

It can be clearly seen in Supplementary Figure 4a that the degree of erosion η strongly depends on both ac frequency and amplitude and is much more pronounced for frequencies well below the FMR frequency ($\omega_{\mathrm{FMR}} = 0.1342$ corresponding to 4.13 GHz). In particular, it exhibits a threshold behavior for ac voltage amplitudes exceeding the value $V_{\mathrm{ac}} = 0.124$ V and decreases for increasing ac drive frequency.

In addition, a visual representation of the erosion is reported in lower panels b,c,d,e of Supplementary Figure 4, which correspond to the values of η, ac drive frequency and voltage associated with markers labeled as b,c,d,e in Supplementary Figure 4a.

More specifically, each of these panels reports, in the (ϕ, m_z) plane, the initial magnetization states corresponding to particles which have not escaped the well within $n = 5$ iterates of the stroboscopic map. One can see that rather complex erosion patterns appear in the basin around the stable equilibrium ($\phi = 0, m_z = 0$). Nevertheless, despite the appearance of such complicated basin erosion, for certain frequencies and amplitudes, it is still possible to recognize safe regions surrounding the stable equilibrium (e.g. in panels B, D, E). In general, one can be easily convinced that, in appropriate ranges of ac voltage amplitude and frequency, a safety region around the minimum of system free energy does exist. This region plays the role of a well in which attractive fixed points or attractive periodic trajectories of the stroboscopic map lie. This well is the one from which the concept of escape in ac-driven conditions can be defined.

SUPPLEMENTARY REFERENCES

[1] Gonçalves, A. M. *et al.* Spin torque ferromagnetic resonance with magnetic field modulation. *Appl. Phys. Lett.* **103**, 172406 (2013).

[2] Tulapurkar, A. A. *et al.* Spin-torque diode effect in magnetic tunnel junctions. *Nature* **438**, 339–342 (2005).

[3] Gilmore, R. & Lefranc, M. *The topology of chaos: Alice in stretch and squeezeland* (John Wiley & Sons, 2012).

[4] Cheng, X., Boone, C. T., Zhu, J. & Krivorotov, I. N. Nonadiabatic stochastic resonance of a nanomagnet excited by spin torque. *Phys. Rev. Lett.* **105**, 047202 (2010).

[5] Locatelli, N. *et al.* Noise-enhanced synchronization of stochastic magnetic oscillators. *Phys. Rev. Appl.* **2**, 034009 (2014).

[6] Mizrahi, A. *et al.* Controlling the phase locking of stochastic magnetic bits for ultra-low power computation. *Sci. Rep.* **6**, 30535 (2016).

[7] Gammaitoni, L., Hänggi, P., Jung, P. & Marchesoni, F. Stochastic resonance. *Rev. Mod. Phys.* **70**, 223–287 (1998).

[8] Badzey, R. L. & Mohanty, P. Coherent signal amplification in bistable nanomechanical oscillators by stochastic resonance. *Nature* **437**, 995–998 (2005).

[9] Finocchio, G., Krivorotov, I. N., Cheng, X., Torres, L. & Azzerboni, B. Micromagnetic understanding of stochastic resonance driven by spin-transfer-torque. *Phys. Rev. B* **83**, 134402 (2011).

[10] Thompson, J. M. T. Chaotic phenomena triggering the escape from a potential well. *Proc. R. Soc. Lond. A. Math. Phys. Sci.* **421**, 195–225 (1989).

[11] d'Aquino, M. *et al.* Chaotic dynamics and basin erosion in nanomagnets subject to time-harmonic magnetic fields. *Phys. B Condens. Matter* **486**, 121–125 (2016).

[12] Ott, E. *Chaos in Dynamical Systems* (Cambridge University Press, Cambridge, 2002).
